# Root Canal Morphology of Mandibular First Permanent Molars in an Indian Population

**DOI:** 10.1155/2012/745152

**Published:** 2012-01-11

**Authors:** Hemant Ramesh Chourasia, Ganesh K. Meshram, Manjusha Warhadpande, Darshan Dakshindas

**Affiliations:** ^1^Department of Conservative Dentistry and Endodontics, Peoples College of Dental Sciences and Research Centre, Bhanpur, Bhopal 462037, India; ^2^Department of Conservative Dentistry and Endodontics, Government Dental College & Hospital, Nagpur 440003, India

## Abstract

An *in vitro* study was performed to determine the number of roots, root canals per tooth, root canal configurations, and frequency of isthmi and apical deltas in mandibular first permanent molars in an Indian population. Hundred and fifty mandibular first permanent molars were collected and subjected to clearing technique. The cleared teeth were examined in a stereomicroscope under 7.5x magnifications. The canal configurations were categorized using Vertucci's classification. Overall 94.6% of the mandibular first molars had two roots, and 5.3% had extradistal roots (distolingual root). In addition, 64% of the specimens had three root canals, and 36% had four root canals. The most common canal configurations of mesial and distal roots were Vertucci type IV (54%) and type I (65.3%), respectively. Clinician should be aware of the complex root canal morphology of mandibular first molars among the Indian population before and during the root canal treatment.

## 1. Introduction

The main goal of endodontics is to restore the function and esthetic of the involved tooth. From a biomechanical perspective, this means cleaning, shaping, and disinfection that would allow for three-dimensional obturation of the root canal system [1]. One of the main reasons for the failure of root canal treatment is the inadequate removal of pulp tissue and microorganisms from the root canal system [2]. It is therefore of utmost importance that the dentist has a thorough knowledge of root canal morphology of the tooth being treated. The mandibular first molar is a frequently treated tooth and has a wide variety of root canal configurations [3]. Variations in the morphology of the dental pulp are caused by genetic and environmental influences, and there is very definite need for clinicians to be made aware of the frequency of racially determined forms [4]. The major variant in mandibular first molars is the presence of a supernumerary root that can be found distolingually and has a curve at the apex. This macrostructure, first mentioned by Carabelli, is called radix entomolaris, which in general is smaller than distobuccal and mesial roots and can be separate from or partially fused with these other roots [2]. The morphology and buccolingual width of the mesial root allow for intercanal communications and isthmuses. Currently, the isthmus (anastomosis) is defined as a pulpal passageway connecting 2 or more canals in the same root [1].

Studies on the root canal anatomy of mandibular first molar have been performed on several populations [1]. An extensive literature search showed that there is only one study on the root canal morphology of Indian mandibular first molars [5]. The root canal anatomy of Indian teeth by clearing technique has not been studied to date except for studies on premolars and mandibular second molars [6]. Hence, the purpose of this study was to prepare detailed investigation of root canal anatomy of mandibular first molar in an Indian population by using clearing technique and to compare these findings with the published reports of different population.

## 2. Materials and Methods

One hundred and fifty mandibular first molars were collected from the Department of Oral and Maxillofacial Surgery of the Government Dental College and Hospital, Nagpur in India. It was ensured that the teeth belonged to indigenous Indians, and no teeth from other minority ethnicities were included. The collection of every tooth was accompanied by a case record stating and confirming the ethnicity of the patients. Teeth that demonstrated fully formed roots and intact external morphology were selected. The teeth were washed under tap water immediately after extraction and stored in 10% formalin (Qualigens Fine Chemicals, Mumbai, India). The residual soft tissues, bone fragments, and calculus were cleansed and removed by curettes and ultrasonic scalers. Each specimen was first examined visually and categorized by the number of roots.

Access cavities were prepared using no. 2 diamond round bur. The specimens were then placed in 5.25% sodium hypochlorite (Prime Dental Products Pvt Ltd., Mumbai, India) for 24 hours in order to dissolve organic debris and pulp remnants. The teeth were then rinsed under running tap water for 2 hours.

The specimens were first decalcified at room temperature in 5% nitric acid (Qualigens Fine Chemicals, Mumbai, India) that was changed daily for 3-4 days. The acid was agitated thrice daily with glass rod, and the end point of decalcification was determined by periodic radiographs. After completion of decalcification, the specimens were washed under running tap water for 4 hours to remove traces of nitric acid. The specimens were dehydrated using ascending concentrations of ethyl alcohol (Thermo Fisher Scientific India Pvt Ltd., Mumbai, India) starting with 70% for 12 hours, followed by 90% for an hour and 3 rinses of 1 hour each for 100%. The dehydrated specimens then were placed in methyl salicylate (Rankem Fine Chemicals Ltd., New Delhi, India) which made them transparent after approximately 2 hours.

Hematoxylin dye (Thermo Fisher Scientific India Pvt Ltd., Mumbai, India) was injected into the pulp chamber with a 27-gauge needle on disposable syringe. The dye then was drawn through the canal system by applying negative pressure to the apical end of the tooth with the help of vacuum suction system. The excess ink was removed from the root surface with gauze soaked in 100% ethyl alcohol. The specimens were returned to the methyl salicylate solution until needed. The cleared teeth were evaluated with a stereomicroscope under 7.5x magnifications. The canal configurations were categorized using Vertucci's classification [7] as follows.

1. *Type I*. A single canal extends from the pulp chamber to the apex.
2. *Type II*. Two separate canals leave the pulp chamber and join short of the apex to form one canal.
3. *Type III*. One canal leaves the pulp chamber, divides into two within the root, and then merges to exit as one canal.
4. *Type IV*. Two separate and distinct canals extend from the pulp chamber to the apex.
5. *Type V*. One canal leaves the pulp chamber and divides short of the apex into two separate and distinct canals with separate apical foramina.
6. *Type VI*. Two separate canals leave the pulp chamber, merge in the body of the root, and redivide short of the apex to exit as two distinct canals.
7. *Type VII*. One canal leaves the pulp chamber, divides and then rejoins within the body of the root, and finally redivides into two distinct canals short of the apex.
8. *Type VIII*. Three separate and distinct canals extend from the pulp chamber to the apex.

The specimens were photographed to provide permanent visual demonstration of their root canal system. The specimens were vertically sectioned in buccolingual direction through the furcation, to separate the mesial and distal halves. Sectioning was done to avoid the overlapping of mesial and distal or distolingual roots during photography.

## 3. Results

The results of this study are summarized in (Tables 1, 2 and 3). Of the 150 mandibular first molars, 94.6% had two roots, and 5.3% had extra distal roots (distolingual root or radix entomolaris). In addition, 64% of the cases had three root canals (mesiobuccal, mesiolingual and distal), and 36% had four root canals (mesiobuccal, mesiolingual, distobuccal and distolingual) (Table 1). 

**Figure 1:**
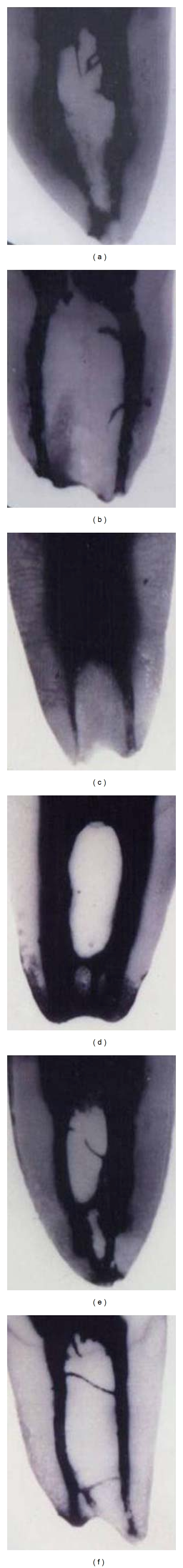
Mesial root canal configurations observed in this study. (a) type II, (b) type IV, (c) type V, (d) type VI, (e) type (2-1-2-1), and (f) isthmi in the cervical and apical third of the root.

**Figure 2:**
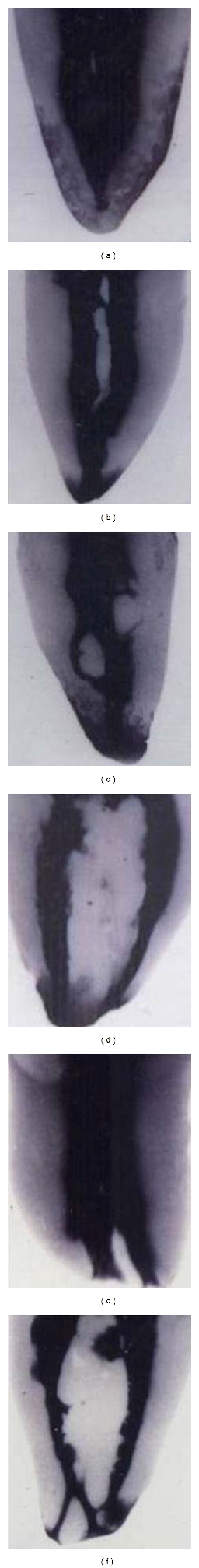
Distal root canal configurations observed in this study. (a) type I, (b) type II, (c) type III, (d) type IV, (e) type V, and (f) apical delta.

Both the mesial and distal roots showed wide variations in canal configuration. In mesial root, type IV configuration was most prevalent (54%) followed by type II (36.6%), type VI (8%), and type V (0.6%) (Figure 1(a)–1(d)). One mesial root showed an additional configuration, Gulabivala et al. [8] type (2-1-2-1) (Figure 1(e)). In distal root, type I configuration was most prevalent (65.3%) followed by type II (20.6%), type IV (9.3%), type V (3.3%), and type III (1.3%) (Figure 2(a)–2(e)). In the three rooted molars, all distolingual roots possessed type I (100%) canal configuration (Table 2).

Isthmi (intercanal communications) (Figure 1(f)) were found in 30% of the cases in mesial and 10% of the cases in distal roots. Apical deltas (Figure 2(f)) were found in 10% of the cases in mesial and 6% of the cases in distal roots (Table 3).

## 4. Discussion

The methods used to study root canal morphology are replication technique [3, 9], clearing technique [6–8, 10–15], use of radiopaque dyes and radiographs [2, 16–20], sectioning of teeth [21], and recently, spiral computed tomography (SCT) [5] and cone beam computed tomography (CBCT) [22]. The clearing technique is most commonly used because of its accuracy. It provides a three-dimensional view of the root canal system. Additionally, it is not necessary to gain an access into the specimen with instruments, thus original form and relationship of the canals are maintained. Hence, clearing technique was used in this study. The clearing technique is simple, but there are some potential problems. These include development of opaque areas due to incomplete dehydration, which can be correctable by additional dehydration in 100% ethyl alcohol. Another common problem is the development of opacity after air drying; however, this is readily reversible by immersion in methyl salicylate solution.

In the present study, the prevalence of three rooted mandibular first molars among the Indian population was 5.3%, similar to the study of Garg et al. [2]. This figure is lower than several earlier studies [8, 14, 16, 22], but higher than that reported by Skidmore and Bjorndal [3] for Caucasians, Zaatar et al. [19] for Kuwaitis, Sperber and Moreau [21] for Africans, and Al-Qudah and Awawdeh [15] for a Jordanian population.

In this study, it was found that 36% of mandibular first molars had four canals. These results are similar to those of Hartwell and Bellizzi [18], who reported 35.1% of teeth and had four canals. This value is lower than the findings of several earlier authors [14, 15, 20, 22], but higher than that reported by Skidmore and Bjorndal [3], Zaatar et al. [19], Sperber and Moreau [21], Gulabivala et al. [8], and Reuben et al. [5]. Owing to the high percentage of two distal canals, classical triangular access preparation during root canal treatment should be extended towards the distolingual direction in a rectangular form to improve canal identification.

In mesial root, type IV configuration was most prevalent (54%) followed by type II (36.6%), type VI (8%), and type V (0.6%) configuration. This is consistent with the findings of most of the earlier studies [3, 7, 8, 12–15, 17, 21, 22], except the studies by Zaatar et al. [19] and Al-Nazhan [20] which reported type II being the most prevalent followed by type IV. In the present study, one mesial root showed an additional configuration type (2-1-2-1) as described by Gulabivala et al. [8]. Identification, preparation, and obturation of type IV, type II, and type VI are relatively straightforward. However, identification of canals in type V, where the canal further divides within the root, is more difficult. The presence of Gulabivala's type (2-1-2-1) needs extra efforts, because failure to debride and disinfect this complex anatomy might have a direct effect on the treatment outcome. There are published reports indicating the presence of type VIII configuration in the mesial root, with the incidence of 0.2% to 5% [7, 8, 12–15, 22]. But in the present study, none of the samples had three canals in the mesial root.

The most prevalent configuration in the distal root was type I (65.3%) followed by type II (20.6%), type IV (9.3%), type V (3.3%), and type III (1.3%) configuration. In terms of type II and type IV configuration, this figure is lower than the studies of Caliskan et al. [12], Ahmed et al. [13], and Al-Nazhan [20], but higher than that reported by Skidmore and Bjorndal [3], Vertucci [7], Gulabivala et al. [8], and Pineda and Kuttler [17]. The external morphology of the distal root is more rounded than the mesial one and therefore less likely to accommodate two separate canals. However, type III and type V configurations in distal root need extra efforts to negotiate and prepare. One of the two canals, the one most continuous with the large main passage, is usually amenable to adequate enlarging and filling procedure, the preparation and filling of the other canal is often extremely difficult [7]. In the three rooted molars, all distolingual roots possessed type I (100%) canal configuration.

In the present study, isthmi (anastomosis) were observed in 30% of the cases in mesial, and 10% of the cases in distal roots. This figure is lower than several earlier studies [7, 8, 12, 13, 15]. Apical deltas were observed in 10% of the cases in mesial and 6% of the cases in distal roots, which is similar to the findings of Vertucci [7] and Caliskan et al. [12], but higher than that reported by Pineda and Kuttler [17], Gulabivala et al. [8], and Al-Qudah and Awawdeh [15]. The presence of isthmi and apical deltas may be of clinical significance, because it may be difficult to debride and fill these ramifications adequately. The use of sodium hypochlorite, preferably agitated by ultrasonics may help to reach the uninstrumented parts of the root canal system [23]. Furthermore, these ramifications can be more satisfactorily obturated by using some thermoplasticized gutta-percha technique rather than cold lateral condensation of gutta-percha points [24].

## 5. Conclusions

The root canal morphology of 150 Indian mandibular first permanent molars shows higher incidence of four canals (36%) and extradistal roots (5.3%). Therefore, the clinician must always look for a second canal in the distal root of Indian mandibular first molars. The most prevalent canal configuration in the mesial root was Vertucci type IV (54%), and in distal root type I (65.3%). An additional configuration, Gulabivala type (2-1-2-1), was found as a rare entity (0.6%) in mesial root. As isthmi and apical deltas were observed in high percentage in mesial and distal root, efficient delivery and activation of irrigants are more essential. Variations in the number of roots or canals and teeth with unusual root canal configurations have a definite impact on treatment. To achieve long-term success, clinician must use all the armamentaria at their disposal to locate and treat the entire root canal system.

## Figures and Tables

**Table 1:** Number of roots and canals in mandibular first molars.

No. of teeth	No. of roots		No. of canals per tooth		
	2	3	3	4	5
150	142	8	96	54	—
	94.6%	5.3%	64%	36%	—

**Table 2:** Canal configuration and the type of root canal in mandibular first molars.

Group (no. of roots)	Canal configuration (type)								
	I	II	III	IV	V	VI	VII	VIII	2-1-2-1
Mesial root (150)	—	55	—	81	1	12	—	—	1
		36.6%		54%	0.6%	8%			0.6%
Distal root (150)	98	31	2	14	5	—	—	—	—
	65.3%	20.6%	1.3%	9.3%	3.3%				
Distolingual root (8)	8	—	—	—	—	—	—	—	—
	100%								

Canal configurations were categorized using Vertucci's classification (1984) [7].

**Table 3:** Frequency of isthmi and apical deltas in mesial and distal roots.

Group (no. of roots)	Isthmi	Apical deltas
Mesial root (150)	45 (30%)	15 (10%)
Distal root (150)	15 (10%)	9 (6%)
Distolingual root (8)	—	—
